# Molecular and Phenotypic Characterization of *Prototheca* Species Isolates Associated with Bovine Mastitis Cases in Chile †

**DOI:** 10.3390/ani15192869

**Published:** 2025-09-30

**Authors:** Jaime Rodriguez, Paulina Sepúlveda-García, Nivia Canales, Matías Goddard, Carlo Cornuy, Álvaro G. Morales, Luis Collado, Armin Mella

**Affiliations:** 1 Instituto de Bioquímica y Microbiología, Facultad de Ciencias, Universidad Austral de Chile, Valdivia 5090000, Chile; g8.jaime@gmail.com (J.R.); paulina.sepulveda.garcia@gmail.com (P.S.-G.); niviaca.mor@gmail.com (N.C.); goddardaguilar@gmail.com (M.G.); luiscollado@uach.cl (L.C.); 2 Escuela de Graduados, Facultad de Ciencias, Universidad Austral de Chile, Valdivia 5090000, Chile; 3 Lecherías del Sur Spa, Osorno 5290000, Chile; carlo.cornuy@lecheriasdelsur.cl; 4 Instituto de Ciencia Animal, Facultad de Ciencias Veterinarias, Universidad Austral de Chile, Valdivia 5090000, Chile; alvaro.morales@uach.cl

**Keywords:** protothecosis, PCR, RAPD-PCR, mastitis

## Abstract

Background: Bovine mastitis caused by *Prototheca* spp. is the most significant animal disease of algal origin, with an increasing number of cases reported worldwide. Currently, there is no effective treatment, so control requires the culling of infected animals. In Chile, information is limited, and a discrepancy remains in the literature regarding the *Prototheca* species involved in bovine mastitis. Methods: This study aimed to molecularly type and phenotypically characterize *Prototheca* isolates associated with bovine mastitis in Chile. Sixty-six *Prototheca* isolates obtained from individual bovine mastitis milk samples and bulk tank milk samples were analyzed through cytochrome b gene (*cytb*) sequencing, Random Amplified Polymorphic DNA–Polymerase Chain Reaction (RAPD-PCR) analysis, and phenotypic evaluation (morphology, antimicrobial susceptibility, and biofilm formation). Results: Sixty-five isolates were identified as *P. bovis* and one as *P. ciferrii*, marking the first report of the latter in bovine mastitis in Chile. RAPD analysis revealed a high genetic diversity in *P. bovis*. All strains exhibited resistance to the antibiotics tested from the Fluoroquinolone, β-lactam, and sulfonamide groups; however, 100% of the strains showed susceptibility to aminoglycosides, with gentamicin standing out as a potential therapeutic option. Most *P. bovis* strains formed weak (81.5%, 53/65) or moderate (15.4%, 10/65) biofilms, which could favor the persistence of infection. Conclusions: These findings provide novel insights into the molecular and phenotypic characteristics of *Prototheca* spp. in Chile, highlighting the predominance of *P. bovis*, the emergence of *P. ciferri*, and the implications for antimicrobial management and disease control.

## Simple Summary

Bovine mastitis is a significant disease impacting dairy cows worldwide, primarily due to the substantial economic losses it causes. Among the pathogens involved, *Prototheca* spp. has gained increasing attention due to the rising number of protothecal mastitis cases and the associated treatment challenges, often resulting in the culling of affected animals. In Chile, only two outdated studies from 2011 have reported protothecal infections in cattle, underscoring the need for updated data to understand the epidemiology of this infection. This study aimed to characterize *Prototheca* isolates from Chilean dairy cows through molecular identification at the species level and assessment of their genetic diversity, antimicrobial susceptibility, and biofilm formation capacity. A total of 66 *Prototheca* spp. isolates were analyzed, with 65 identified as *P. bovis* and one as *P. ciferrii*. Genetic analysis revealed 18 distinct *P. bovis* genotypes without clustering by farm origin, indicating high genetic heterogeneity. All isolates exhibited resistance to most antibiotics tested except aminoglycosides. Additionally, the isolates demonstrated weak to moderate biofilm production, which may contribute to the persistence of infections. This work represents the first comprehensive study of *Prototheca* in Chile and the third worldwide to assess *P. bovis* genetic diversity, providing valuable insights for the development of alternative therapeutic strategies.

## 1. Introduction

Bovine mastitis is one of the most prevalent and challenging diseases worldwide, causing substantial economic losses due to reduced milk yield and poor quality [1,2]. Unlike many other animal diseases, it can be caused by a wide range of microorganisms, including bacteria, viruses, fungi, and unicellular algae [3]; among them, the genus *Prototheca* has gained increasing importance, being ranked among the major etiological agents of bovine mastitis in countries such as Canada, Italy, and Poland [4,5,6,7]. However, globally, protothecal mastitis remains relatively uncommon [8,9].

The genus *Prototheca* (family Chlorellaceae, order Chlorellales) comprises achlorophyllous microalgae that evolved from the green alga *Chlorella* through the loss of photosynthetic capacity, leading to their adaptation to heterotrophic conditions, which enables them to cause opportunistic infections collectively referred to as Protothecosis in humans and other animals [4]. Recent taxonomic revisions, based on the analysis of the mitochondrial cytochrome b gene (*cytb*), have led to a reclassification of the species of the genus *Prototheca*, providing higher resolution compared to conventional ribosomal DNA (rDNA) markers [10,11]. Consequently, 18 *Prototheca* species have been recognized, among which *P. ciferrii* (formerly *P. zopfii* genotype 1), *P. bovis* (formerly *P. zopfii* genotype 2), *P. blaschkeae*, *P. cutis*, *P. miyajii*, and *P. wickerhamii* have been identified as etiological agents of diseases in humans and animals [4,12]. Protothecal infection in humans generally triggers three main clinical manifestations: cutaneous lesions, olecranon bursitis, or systemic infections, with *P. wickerhamii* associated with cutaneous cases, while *P. bovis* is principally involved in systemic protothecal infection [4].

*P. bovis* is undoubtedly recognized as the principal agent involved in bovine protothecal mastitis [13,14], and the presence of *P. blaschkeae* is occasionally reported [15]. In contrast, the role of *P. wickerhamii* [16,17] and *P. ciferrii* [8] in bovine protothecal mastitis remains unclear [8,18]. Since the first detection of *Prototheca* infection in cattle by Lerche (1952) [19], these pathogens have been reported as emerging pathogens across all continents, causing acute or chronic bovine mastitis with visibly abnormal milk, swelling of the mammary gland, and reduced milk yield, resulting in economic losses [5,7,20]. They may also represent a serious problem due to the inherent resistance of these microalgae to different classes of antimicrobial drugs [14]. Currently, there are no established treatment guidelines for protothecal infections in humans and animals. Due to the similarity in the cell membrane composition of *Prototheca* and yeast (the presence of ergosterol), the treatment of human protothecosis typically includes antifungal agents in combination with surgical approaches [4]. Nevertheless, the administration of these agents in dairy cattle is considered impractical, primarily due to their high economic cost and toxicity to mammary gland tissue [13,21,22].

It is noteworthy that there is no effective treatment for mastitis caused by *Prototheca* spp.; thus, culling infected cows is recommended to prevent the spread of infection to other cows and to avoid unnecessary antibiotic use [23].

Additionally, some authors have noticed the ability of *Prototheca* to produce biofilm, suggesting that it enhances pathogenicity and contributes to antimicrobial resistance and a reduced effectiveness of the immune response [23]. Although most antimicrobials exhibit limited efficacy, some antibiotics and antifungals have demonstrated variable in vitro activity [14,17,24,25].

Current knowledge about the genetic polymorphism of *Prototheca* strains is limited to two studies using Random Amplified Polymorphic DNA (RAPD)-PCR, which have shown significant heterogeneity among the *Prototheca* isolates and highlighted the potential of RAPD-PCR for identifying *P. bovis* strains at the genetic level [14,24].

Despite the growing global importance of protothecal bovine mastitis [8,9], available information remains limited in many countries. Chile is not an exception, as there are only two dated studies from 2011 that reported the first identification of *P. zopfii* associated with bovine mastitis in the country [26], and the genotyping and characterization of the antimicrobial and antifungal susceptibility of *Prototheca* isolates during a bovine mastitis outbreak, revealing that *P. zopfii* genotype 2 was involved in 46.6% (26/58) of the affected animals [27]. Although both studies demonstrated the presence and potential significance of this microalga in cases of bovine mastitis, no subsequent studies have been conducted in Chile. Updated research is necessary to revise the existing information based on the new taxonomic nomenclature, as well as to incorporate advanced techniques for species-level identification and genotyping. This would enable a more accurate identification of the *Prototheca* species and variants currently affecting dairy cattle in Chile, recognizing that the precise identification of *Prototheca* species is crucial, as they differ in their pathogenicity and antimicrobial sensitivity patterns [17,24]. This study aims to perform molecular typing and phenotypic characterization of *Prototheca* isolates associated with bovine mastitis cases in Chile. The approach involves species identification, analysis of genetic diversity using RAPD-PCR, and phenotypic characterization, which encompasses morphological analysis, antimicrobial sensitivity testing, and assessment of biofilm formation capacity. This comprehensive approach aims to provide relevant information on the epidemiology of protothecal mastitis in Chilean dairy herds and contribute to the design of more effective control strategies.

## 2. Materials and Methods

### 2.1. Sample Origin

A convenience sampling study was performed for the analysis of milk samples collected from animals with clinical or subclinical bovine mastitis that arrived at the Bovine Mastitis Laboratory of the Universidad Austral de Chile (UACh), Valdivia, Chile, between January 2023 and October 2024. A total of 6442 individual or composite milk samples from 8 dairy farms in Southern Chile (farms A, B, C, D, E, F, G, H) and 104 bulk tank milk samples from 7 farms in Central and Southern Chile were analyzed (farms H, I, J, K, L, M, N). Additionally, 58 milk samples from cows affected by an outbreak of clinical mastitis refractory to treatment that occurred in January 2010 at a dairy farm in the metropolitan region of Central Chile (farm O) were included in this study.

### 2.2. Prototheca spp. Isolation

For *Prototheca* spp. isolation, 0.1 mL of milk samples was plated on 5% sheep blood agar, while samples from bulk tanks were plated on Sabouraud dextrose agar supplemented with chloramphenicol (100 mg/mL). The plates were incubated aerobically at 37 °C for 48–72 h, following the National Mastitis Council (NMC) guidelines [28].

The macroscopic morphology (shape, size, color, and opacity) of the colonies was analyzed under a stereomicroscope. The resulting *Prototheca* suspicious colonies (cream-white, pasty colonies) were examined through Gram-stain and wet mounts with lactophenol blue using optical microscopy. According to the NMC, the presence of spherical-oval sporangia, with or without endospores, is considered indicative of *Prototheca* [28]. All *Prototheca* spp. suspected colonies were cryopreserved in liquid *Prototheca* isolation medium (PIM) with glycerol 20% for subsequent analyses. Additionally, two reference strains were included: *P. bovis* SAG 2021^T^ and *P. ciferrii* SAG 2063^T^, acquired from the Culture Collection of Algae (SAG) at the University of Göttingen, Germany.

### 2.3. Molecular Identification of Prototheca Species

#### 2.3.1. DNA Extraction and Amplification of *Prototheca* spp. *Cytb* Partial Gen

Genomic DNA was extracted with modifications using a Cetyltrimethylammonium Bromide (CTAB)-based protocol [29] in which an additional lysis step was incorporated. This step involved mechanical lysis followed by chemical lysis with SDS/Proteinase K to facilitate disruption of the *Prototheca* wall cell. Briefly, three to five colonies were resuspended in a microcentrifuge tube containing 400 μL of 1× TE buffer and 0.5 mm zirconia/silica beads (BioSpec Products) and were subjected to mechanical lysis by agitation in a microtube homogenizer (Mini BeadBeaster-16, BioSpec Products) for 1 min. Subsequently, the suspension was digested with SDS/Proteinase K at 65 °C for 10 min. DNA extraction was performed with a chloroform/isoamyl alcohol mixture (24:1), followed by DNA precipitation with isopropanol. The pellet was washed with 70% ethanol, dried, and resuspended in TE buffer. DNA Samples were stored at −20 °C for subsequent analyses.

To identify *Prototheca* spp. at the level of species, a PCR was performed as previously described by [11]. Briefly, a partial fragment (644 bp) of the *cytb* gene was amplified using the primers cytb_F1 (5′-GYGTWGAACAYATTATGAGAG-3′) and cytb_R2 (5′-WACCCATAARAARTACCATTCWGG-3′) in a reaction mixture (50 μL) containing 25 μL of Promega GoTaq^®^ DNA polymerase (Promega, Madison, WI, USA), 0.2 μM of each primer, 18 μL of Milli-Q ultrapure water, and 5 μL of template DNA, under the thermal protocol consisting of: initial denaturation at 95 °C for 3 min followed by 35 cycles of denaturation at 95 °C for 30 s, annealing at 50 °C for 30 s, and extension at 72 °C for 30 s, with a final extension at 72 °C for 5 m in a SureCycler 8800 thermocycler (Agilent, Santa Clara, CA 95051, USA) [11,13]. PCR products were visualized by electrophoresis on 1% (*w*/*v*) agarose gel stained with SYBR Safe (Invitrogen, Carlsbad, CA 92008, USA). The amplicons were sent for purification and sequencing to Macrogen Chile (Santiago, Chile), where they were sequenced by the Sanger method in both directions using the same PCR primers.

#### 2.3.2. Phylogenetic Analysis

Initial sequence analysis was performed using Geneious Prime 2025.2 [30] to assess sequence quality, trim the sequence, and generate the consensus sequence. The resulting *cytb* gene sequences were analyzed using the BLASTn tool [31] to determine the identity percentage compared with sequences in the GenBank database. Subsequently, the sequences obtained in the present study, together with sequences retrieved from GenBank, were aligned using the MAFFT (Multiple Alignments by fast Fourier Transform) method [32] and analyzed using BMGE (Block Mapping and Gathering with Entropy) software to remove ambiguously aligned regions [33]. The same procedure was performed, involving only the sequence of the present study to estimate the identity distance among them, generating a distance matrix. From those sequences with 100% identity, a single sequence was randomly selected and submitted to GenBank [34].

Two phylogenetic trees were constructed based on two different phylogenetic inference methods: Maximum Likelihood (ML) and Bayesian Inference (BI). Prior to the ML phylogenetic tree inference, the best nucleotide substitution model was selected according to the Bayesian Information Criterion (BIC) [35] for each codon position (partition) [36] using ModelFinder [37]. Thereafter, the tree was inferred by the ML method on IQ-TREE [38,39]. The topology’s robustness was evaluated with 1000 bootstrapping replicates, employing the rapid-hill-climbing and stochastic disturbance methods with IQ-TREE [38,39].

For phylogenetic construction based on the BI method, the appropriate nucleotide substitution models were independently selected for each partition using the Reversible Jump Markov Chain Monte Carlo (MCMC) algorithm [40]. Then, the phylogenetic tree was inferred by running 20,000 generations in Mr. Bayes version 3.2 available on CIPRES [41]. Bayesian posterior probabilities (BPP) with values ≥ 0.70 were considered to provide strong statistical support [40]. Finally, a consensus tree (among the ML and BI resulting phylogenetic construction) was generated using the Consensus Tree Builder in Geneious Prime 2025.2 [30].

### 2.4. RAPD-PCR Genotyping

The genetic diversity of *Prototheca* spp. isolates was analyzed using the molecular marker Random Amplified Polymorphic DNA-Polymerase Chain Reaction (RAPD-PCR). The study also included DNA from reference strains *P. bovis* SAG 2021^T^ and *P. ciferrii* SAG 2063^T^.

Based on previous RAPD-PCR studies [14,24], the OPA-4 primer (5′-AATCGGGCTG-3′) was selected for the present analysis due to evidence of its ability to generate distinguishable band patterns for genetic discrimination among the isolates [14]. Amplification was carried out under the following conditions: initial denaturation at 94 °C for 2 min, followed by 55 cycles of 94 °C for 1 min, 40 °C for 2 min, and 72 °C for 2 min, with a final extension at 72 °C for 5 min. The dendrogram was generated using Phoretix 1.0 software and the Unweighted Pair Group Method with Arithmetic Mean (UPGMA) clustering method. According to Morandi et al. (2016) [24], strains with a similarity coefficient equal to or greater than 90% can be considered genotypically identical.

### 2.5. Antibiotic Susceptibility

The antibiotic susceptibility evaluation of *Prototheca* spp. isolates were performed using the disk diffusion method on Müller-Hinton agar (Liofilchem, Roseto degli Abruzzi, Italy), in triplicate, following the guidelines of the Clinical and Laboratory Standards Institute (CLSI) [42]. The panel of antibiotics (Oxoid, Basingstoke, UK) evaluated comprised representatives of different families: gentamicin (CN) (10 μg), netilmicin (NET) (30 μg), kanamycin (K) (30 μg), colistin sulfate (CT) (50 μg), lincomycin (MY) (2 μg), ciprofloxacin (CIP) (5 μg), enrofloxacin (ENR) (5 μg), amoxicillin (AML) (25 μg), penicillin (P) (10 IU), ampicillin (AMP) (10 μg), and sulfamethoxazole/trimethoprim (SXT) (25 μg). The plates were incubated at 37 °C for 48 h, after which the diameters of the growth inhibition zones (mm) were measured. Since there are no specific standardized criteria for interpreting susceptibility tests in *Prototheca* spp., the classification system previously proposed by Morandi et al. (2016) [24] was adopted, categorizing strains according to the inhibition halo diameter as: susceptible (≥9 mm), intermediate (3–8 mm), and resistant (≤2 mm). The reference strain *Escherichia coli* ATCC 25923 was used as a control.

### 2.6. Biofilm Formation

Biofilm formation was evaluated following the methodology described by Morandi et al. (2016) and Tashakkori et al. (2022) [14,24]. Briefly, *Prototheca* spp. cell suspensions were prepared in Sabouraud dextrose broth (SDB) (BD Difco^TM^, Le Pont de Claix, France) and incubated for 48 h at 37 °C. In 96-well flat-bottom cell culture plates, 200 μL of the culture was inoculated and diluted in a 1:9 ratio in SDB. The assays were performed in triplicate, including negative controls with SDB. After incubating the plates without agitation at 37 °C for 24 h, the medium was removed by aspiration, and the wells were washed with PBS. Following the established protocol, the plates were dried at 45 °C for 3 h, stained with 200 μL of 2% crystal violet for 20 min., rinsed with sterile water, and dried at room temperature. The dye adhered to the biofilm was solubilized with 200 μL of 33% acetic acid. Absorbance readings were quantified at 490 nm (OD490) in a Synergy^TM^ 2 microplate reader (Bio Tek, USA). The ability of strains to produce biofilm was classified as weak (OD NC < OD ≤ 2 × OD NC), moderate (2 × OD NC < OD ≤ 4 × OD NC), or strong (OD > 4 × OD NC), where OD NC is the optical density of the negative control.

### 2.7. Statistical Analysis

Data were analyzed according to the type of variable assessed. Antimicrobial susceptibility and biofilm formation results were summarized using descriptive statistics, reporting categorical outcomes, frequencies, and percentages. Genotyping analysis by RAPD-PCR was interpreted through similarity coefficients and UPGMA clustering, applying a ≥90% threshold to define identical profiles. For phylogenetic inference, Maximum Likelihood and Bayesian approaches were used, and node support was evaluated with 1000 bootstrap replicates and Bayesian posterior probability values, considering thresholds ≥ 0.70 as strong statistical support. No additional inferential analyses were applied, as the study was intended to provide a descriptive characterization of the isolates.

## 3. Results

### 3.1. Prototheca Isolation

**Figure 1:**
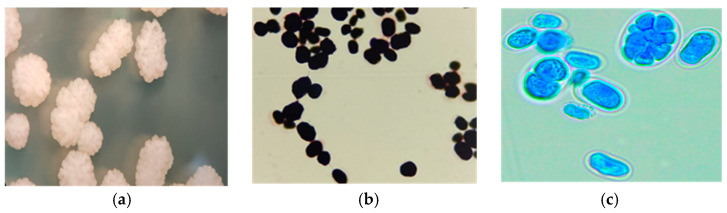
Morphological characteristics of *Prototheca* spp. (**a**) Colonies on SDA observed under a stereomicroscope, appreciating the whitish coloration, pronounced elevation, and a cauliflower-like shape. (**b**) Gram-stain (100×) of *Prototheca* cells that are completely purple staining (internal structures are not distinguishable). (**c**) Preparation with lactophenol blue (100×). Sporangial-type cells (characteristic of *Prototheca*) containing 2 to 8 or more sporangiospores and a well-defined cell wall are observed.

The identification of positive *Prototheca* culture was initially achieved based on the macroscopic characteristics of the colonies observed under a stereomicroscope, as well as microscopic observations of the cells using Gram-staining and wet mount preparation with lactophenol [28]. The macroscopic examination revealed colonies with irregular morphology, whitish coloration, shiny appearance, pronounced elevation, wavy edge, and granular surface (mulberry or cauliflower-like) (Figure 1a). The microscopic evaluation showed oval, spherical, or reniform structures, individual cells or small groups stained purple with Gram-staining (internal structures are not evidenced) (Figure 1b) and blue in wet preparation with lactophenol, showing sporangial-type cells (characteristic of *Prototheca*) containing 2 to 8 or more sporangiospores and an evident cell wall (Figure 1c).

Was observed a culture positivity occurrence of the culture of 0.36% (23/6442) in individual or composite milk samples from dairy farms in southern Chile; 15.38% (16/104) in bulk tank milk samples from 7 farms in Central and Southern Chile; and 46.55% (27/58) in an outbreak of clinical mastitis in a farm of Metropolitan Region of Chile (farm O). Thus, a total of 66 *Prototheca* spp. isolates were analyzed from individual milk samples (50 isolates) and bulk tank milk samples (16 isolates) (Table A1).

### 3.2. Molecular Identification of Prototheca Species

#### 3.2.1. DNA Extraction and Amplification of *Prototheca* spp. Cytb Partial Gene

All *Prototheca* spp. suspected isolates (n = 66) showed a positive amplification of the *cytb* partial gene, reflected in the presence of the expected size band (644 bp), resulting in 66 PCR products successfully sequenced. The BLASTn analysis of the obtained sequences showed that 65 sequences exhibited high identity percentages (98.52 to 100%) with *P. bovis cytb* gene detected in bovines from Germany (MF163469), Japan (AP038925), Spain (MZ604423, MZ404428), China (OP748363), and *P. bovis* from a dog in Australia (MT240530). Based on the estimation of distance among the 65 *cytb-Prototheca* spp. sequences, six genetic variants were observed and deposited in GenBank under the accession numbers PV768537-42 (Table A2). One of the sequences showed 100% identity with *P. ciferrii* identified in a human from Puerto Rico (MK452796) and was deposited in GenBank under the accession number PV768543 (Table A2).

#### 3.2.2. Phylogenetic Analysis

**Figure 2:**
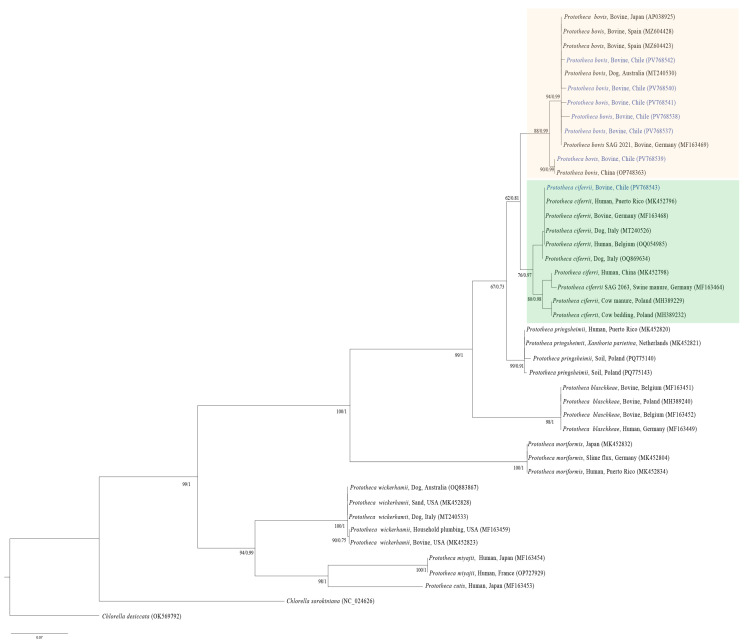
Consensus Maximum likelihood (ML) and Bayesian Inference (BI) phylogenetic tree based on alignment of 594 bp of the partial *cytb* gene of a subset of *Prototheca* spp. sequences. The best substitution models estimated for each one of the codon positions (partition) were HKY+F+G4 for partitions 1 and 2, and TIM+F+G4 for partition 3, based on Bayesian Information Criterion (BIC) for the ML inference method. The selected substitution models using the Reversible Jump Markov Chain Monte Carlo (MCMC) algorithm were: M15, M90 for partition 1; M20, M107 for partition 2; and M88, M150, M185, M197, M187 for partition 3, for the phylogenetic construction inferred by BI. The green box indicates the clade comprising the *P. ciferrii* sequence isolated in this study, together with *P. ciferrii* sequences obtained from the database. Similarly, the beige box frames the clade corresponding to *P. bovis*.

The resulting phylogenetic trees from the BI and ML methods supported the BLASTn analysis outcomes. The consensus tree showed that the sequences of the present study (PV768537-43) were allocated into two distinct clades, one that grouped *P. bovis* sequences and the other comprising *P. ciferrii*. Within the *P. bovis* group, most of the *P. bovis* sequences clustered closely together with *P. bovis* originating from Germany, Australia, Japan, and Spain, except for sequence PV768539, which was placed in a different clade together with *P. bovis* from China. In contrast, the sequence PV768543 was placed in the *P. ciferrii* clade, closely related to *P. ciferrii* from a dog in Italy, a bovine in Germany, and humans from Puerto Rico and Belgium (Figure 2).

### 3.3. RAPD-PCR Genotyping

**Figure 3:**
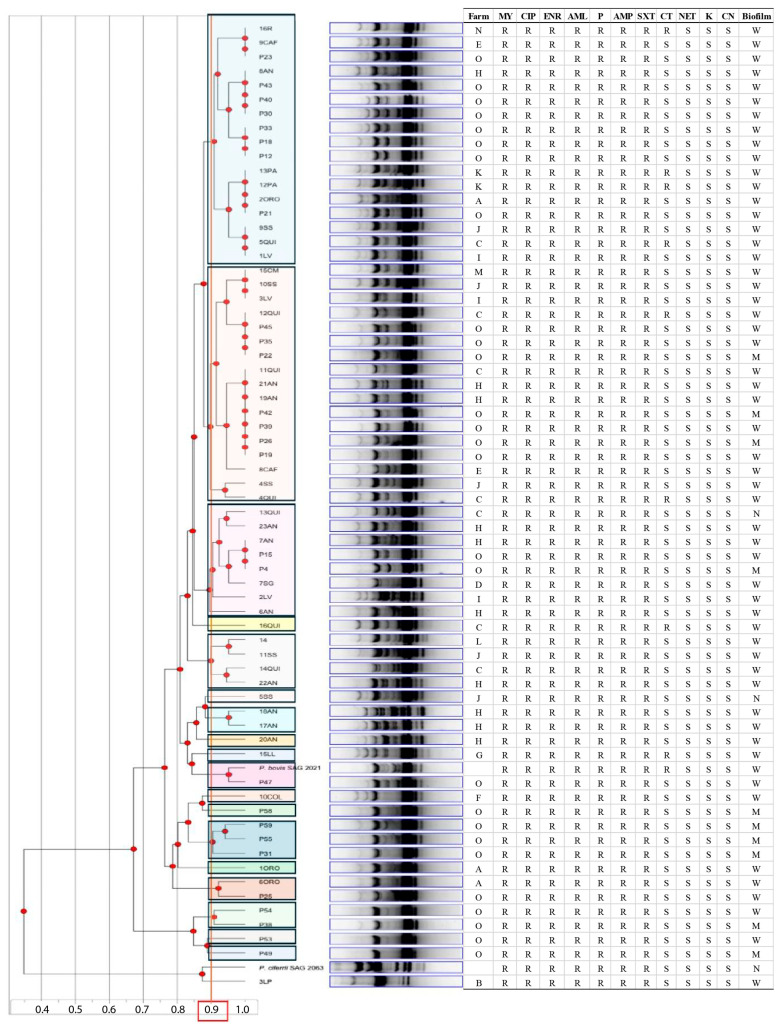
Dendrogram obtained from the amplification profiles generated with the OPA-4 primer, using the Unweighted Pair Group Method with Arithmetic Mean (UPGMA). Strains with a similar coefficient equal to or greater than 90% can be considered genotypically identical (clustered within the same color) [24]. The analysis includes the *Prototheca* isolates studied, along with the reference strains *Prototheca bovis* SAG 2021^T^ and *Prototheca ciferrii* SAG 2063^T^. The information for each isolate is indicated: farm, susceptibility (R: resistant and S: susceptible) to tested antibiotic (MY: lincomycin, CIP: ciprofloxacin, ENR: enrofloxacin, AML: amoxicillin, P: penicillin, AMP: ampicillin, SXT: sulfamethoxazole/trimethoprim, CT: colistin sulfate, NET: netilmicin, K: kanamycin, CN: gentamicin) and biofilm production (W: weak; M: moderate; N: nonproduction).

The RAPD-PCR analysis with the OPA-4 primer generated band profiles that enabled the evaluation of genetic diversity among the 66 *Prototheca* spp. isolates, together with the two reference strains. The resulting dendrogram, constructed using the UPGMA method, revealed that the 65 *P. bovis* isolates were sorted into 18 different genotypes with a distribution independent of the origin farm (Figure 3).

The analysis successfully grouped the isolates according to their species. The 3LP isolate clustered with the type of strain *P. ciferrii* SAG 2063^T^, confirming its identity as *P. ciferrii*. The remaining isolates clustered with the type of strain *P. bovis* SAG 2021^T^, indicating their assignment to this species.

### 3.4. Antibiotic Susceptibility

**Figure 4:**
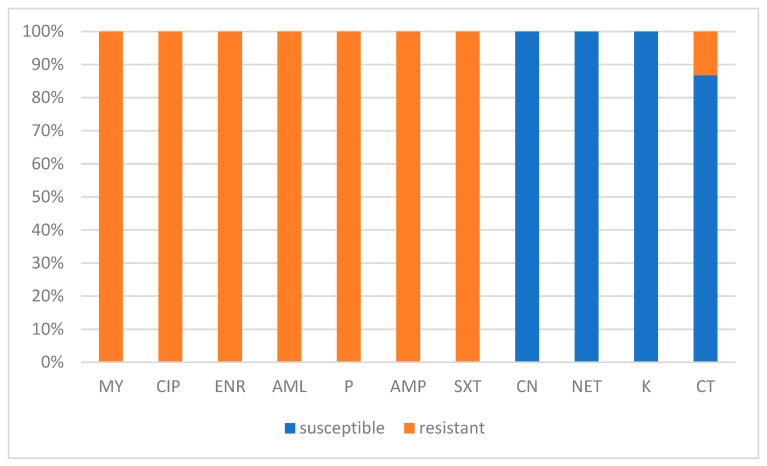
Percentage of *Prototheca* isolates classified as susceptible or resistant to antibiotics tested in this study. CIP: ciprofloxacin, ENR: enrofloxacin, AML: amoxicillin, P: penicillin, AMP: ampicillin, SXT: sulfamethoxazole/trimethoprim, CN: gentamicin, NET: netilmicin, K: kanamycin, CT: Colistin sulfate.

All isolates showed resistance to lincomycin (MY), ciprofloxacin (CIP), enrofloxacin (ENR), amoxicillin (AML), penicillin (P), ampicillin (AMP), and sulfamethoxazole/trimethoprim (SXT), with no inhibition zones observed (inhibition diameter = 0 mm). Conversely, gentamicin (CN), netilmicin (NET), and kanamycin (K) exhibited the highest antimicrobial activity, with all strains classified as susceptible. Colistin sulfate (CT) showed variable activity, with 13.24% (9/68) of the isolate exhibiting resistance. No intermediate susceptibility was observed for any of the antibiotics tested (Figure 4). The antibiotic susceptibility results of the isolates appeared independent of their RAPD-genotype or farm origin. The individualized antibiotic susceptibility outcomes by isolate are detailed in Figure 3.

### 3.5. Biofilm Formation

It was observed that the majority of *P. bovis* isolates in the present study were classified as weak biofilm producers (81.5%, 53/65). A moderate biofilm production was observed in 15.4% (10/65) of the isolates, while only two isolates (3.1%) showed no evidence of biofilm formation. The reference strains, *P. bovis* SAG 2021^T^, showed weak biofilm production. The only *P. ciferrii* isolate obtained in the present study exhibited weak biofilm production, whereas the reference strain *P. ciferrii* SAG 2063^T^ was classified as a non-producer (Table 1).

## 4. Discussion

The present study revealed the identification of *P. bovis* as the principal species involved in bovine protothecal mastitis cases in different farms from Central and Southern Chile, identifying 65 of the 66 isolates as *P. bovis* through molecular techniques, which is consistent with previous studies that have identified this species as the main etiological agent in several regions of the world. The isolation of a *P. ciferrii* strain from a quarter of a cow with subclinical mastitis, without detecting any other mammary pathogens, suggests that *P. ciferrii* could be a potential causal agent of mastitis. However, this idea should be approached with caution since most studies have demonstrated that the isolation of *P. ciferrii* from mastitis cow milk samples is unusual [5,7,25,43,44], and it has been isolated more frequently from cow barn surroundings. Consequently, it is proposed that *P. ciferrii* is not involved in bovine mastitis [25,43]. Furthermore, the presence of *P. ciferrii* in stool and rectal swabs from healthy dairy cows suggests its role as part of the non-pathogenic microbiota of the bovine gut [7]. Nonetheless, a few studies have challenged the suggestion of non-pathogenicity, reporting the isolation of *P. ciferrii* from individual milk samples from cows with mastitis from different family dairy herds in Italy [8]. Additionally, mammary gland tissue from a cow experimentally infected with *P. ciferrii,* as analyzed histopathologically, exhibited the development of typical features of mastitis similar to those induced by *P. bovis* [18]. Recent studies have also documented its presence in human [45] and dog [46,47] infections. In this context, this study represents the second global record of *P. ciferrii* presence in milk samples from bovine mastitis cases and the first reported in Chile. However, the potential pathogenicity of *P. ciferrii* remains controversial, warranting further research to elucidate its role in bovine mastitis.

The analysis of genetic diversity by RAPD-PCR in 66 *Prototheca* spp. strains enabled the evaluation of genotypic variability within the Chilean population of this genus. The dendrogram generated with the OPA-4 primer, clustered using the UPGMA method, clearly separated the two species (*P. bovis* and *P. ciferrii*), confirming the utility of this technique for inter-specific differentiation within *Prototheca*. These findings are consistent with those reported by Morandi et al. (2016) [24], who observed a grouping of strains according to their species [24].

Regarding intra-specific diversity, the classification of the *P. bovis* isolates into 18 different genotypes was observed, evidencing the genetic heterogeneity of this species. Similar results were reported in two previous studies [14,24]. One of these studies identified 28 distinct genotypes of *P. bovis* [24], while the other found that only four pairs of isolates were genotypically identical among 48 *P. bovis* isolates [14]. Additionally, the dendrogram created from the RAPD-PCR banding patterns showed that the genotype distribution did not correlate with the farm of origin, antibiotic susceptibility, or biofilm production. Indeed, the isolates obtained from the protothecal mastitis outbreak were clustered indiscriminately. To the best of the authors’ knowledge, only two studies to date have explored intraspecific genetic variation in *Prototheca* spp. using RAPD-PCR, which limits the possibility of drawing conclusive comparisons. One of these studies [14] reported similar findings, revealing high genetic heterogeneity: only four pairs of *P. bovis* isolates were genotypically identical out of 48 tested using the OPA-4 primer. Moreover, the technique was unable to cluster the resulting genotypes based on farm origin, antibiotic susceptibility, or biofilm production. The study by Morandi et al. (2016) [24] also aligns with the present findings regarding the genetic variability of *P. bovis*, identifying 28 distinct genotypes. However, unlike the present study, which used only the OPA-4 primer, Morandi et al. (2016) differentiated strains based on geographical origin by analyzing the combined banding profiles generated by three primers (M-13, OPA-4, and OPA-18) and comparing isolates from two different countries (Brazil and Italy). It has been reported that combining multiple primers increases the discriminatory power of RAPD-PCR. For example, Idil & Bilkay (2014) [48] found that some strains from different hospitals could not be distinguished using a single primer. Therefore, the use of multiple primers is recommended to enhance the discriminatory resolution of RAPD-PCR and potentially enable the differentiation of isolates according to their origin. Additionally, future studies should consider incorporating high-resolution methodologies, such as sequencing-based molecular markers, to allow a more comprehensive characterization of the *P. bovis* population structure in Chile.

The antimicrobial susceptibility profile of *Prototheca* spp. strains examined in this study showed broad resistance to most antibiotics tested, except for aminoglycosides and, to a lesser degree, polymyxins. These results align with previous research indicating significant resistance of *Prototheca* spp. to conventional antibiotics, especially β-lactams, fluoroquinolones, and Sulfonamides/Trimethoprim [49,50]. β-lactams work by blocking bacterial cell wall synthesis through inhibiting transpeptidases, preventing the transformation of immature peptidoglycan into its mature, functional form [51]. Fluoroquinolones target bacterial DNA gyrase and topoisomerase IV, enzymes vital for DNA replication [51]. Sulfonamides and trimethoprim inhibit the synthesis of tetrahydrofolic acid, the active form of folic acid necessary for the synthesis of thymidine, purines, and ultimately DNA [52].

These mechanisms are crucial for the survival of most prokaryotic organisms. However, they are absent or significantly different in eukaryotic cells, supporting the hypothesis that *Prototheca* spp., as eukaryotic algae, lack specific cellular targets for these antibiotics, therefore possess an intrinsic resistance mechanism to these antimicrobial classes. This is a noteworthy consideration, as the indiscriminate use of antibiotics in dairy cattle can contribute to the development of antimicrobial resistance. This highlights the importance of raising awareness about *Prototheca* spp. as a causative agent of bovine mastitis. In many cases, bovine mastitis (including *Prototheca* spp.) is treated with antibiotics without an etiological diagnosis, which are ineffective against *Prototheca* spp. Consequently, such misdiagnosis often leads to refractory cases and unnecessary antibiotic use [4].

On the other hand, aminoglycosides proved to be the most effective antibiotics, with all strains found to be susceptible to gentamicin, kanamycin, and netilmicin. This finding is consistent with previous studies that have reported high susceptibility of *Prototheca* spp. to aminoglycosides [14,24,25,49,53]. Furthermore, in a murine model of protothecal mastitis, administration of gentamicin at doses of 20–30 mg/kg significantly reduced *Prototheca* load in mammary glands, supporting its potential therapeutic use for *Prototheca* infections [54]. This finding is particularly relevant given the availability of commercial formulations of gentamicin, both parenteral and intramammary, approved for use in cattle in veterinary practice. The convergence of in vitro and in vivo results suggests that gentamicin could be considered as a promising therapeutic alternative for treating bovine protothecal mastitis. However, controlled clinical trials in cattle with protothecal mastitis are necessary to validate its efficacy and establish optimal treatment protocols.

Colistin sulfate showed variable activity, with 13.2% of strains found to be resistant. This result is comparable to the findings of Tashakkori et al. (2022) [14], who reported 89.58% susceptibility to colistin, suggesting that this antibiotic may play a role in controlling *Prototheca* spp. infections depending on the strain and clinical context. This heterogeneity in the response to polymyxins has also been reported by Wawron et al. (2013) [50], who found that 29.6% of *Prototheca* strains exhibited intermediate susceptibility to colistin.

Regarding the biofilm production observed in *P. bovis* isolates of the present study, most of the isolates (81.5%) exhibited weak biofilm-forming capacity, while 15.4% showed moderate production, and only 3.1% did not display this ability. Unfortunately, to the best of our knowledge, studies that assess *Prototheca* spp. biofilm production is limited and modified from procedures created to assess biofilm production in bacteria; thus, it is necessary to generate a standardized assay to evaluate biofilm production in this microalga [14,24,55]. The outcomes obtained in the present study are similar to those reported by Gonçalves et al. (2015) [55], with most isolates classified as weak biofilm producers, followed by moderate biofilm producers. Otherwise, discrepancies in the degree of production among studies have been noted. Morandi et al. (2016) [24] reported that a remarkable percentage of *P. bovis* isolates exhibit strong production at 37 °C, whereas Tashakkori et al. (2022) [14] found a higher proportion of non-producing strains (41.66%), followed by moderate and weak producers (25% each). These inconsistencies among studies could be partially explained by differences in *Prototheca* spp. strain, Morandi et al. (2016) [24] suggested that the ability to produce biofilm is strain-dependent.

Regarding *P. ciferrii*, our field isolate exhibited weak biofilm production, while *P. ciferrii* SAG 2063^T^ did not form biofilms. This observation partially agrees with Morandi et al. (2016) [24], who classified the *P. ciferrii* reference strain as a weak producer, supporting previous reports on this species’ lower pathogenicity [43,56].

It is noteworthy that all moderate biofilm producers isolates originated from a mastitis outbreak, suggesting a possible role of biofilm in *Prototheca* infections, as mentioned by Kwiecinski (2015) [23], who concluded that biofilm production property could contribute to promoting *Prototheca* infection, partly by preventing IL-6 signaling and the initiation of the immune response.

The biofilm-forming ability in 96.9% of our strains, although predominantly weak, suggests that this trait may be relevant to the pathogenesis of *P. bovis*-associated mastitis in Chilean herds. According to Kwiecinski (2015) [23], this property may contribute to the chronicity of the infection and to resistance against antimicrobial treatments, as well as to the host immune response factors frequently associated with protothecal mastitis. In a nutshell, studies on *Prototheca* spp. biofilm formation are scarce; nonetheless, the available reports noticed the ability of *Prototheca* strain to produce biofilm in variable degrees, highlighting their role in the persistence of this alga in sustaining infection [14,24,55]. Nevertheless, further research is needed to elucidate the association between biofilm production and the complexity of clinical manifestation, as well as its impact on disinfection procedures and milk pasteurization. These aspects are particularly relevant for public health, as they involve understanding the zoonotic potential of this alga and the possibility of transmission through the consumption of contaminated milk or dairy products [8,9].

## 5. Conclusions

The present study confirmed the relevance of *P. bovis* as the principal species involved in l mastitis cases in Chile and reports the first identification of *P. ciferrii* in mastitis cow’s milk samples. Additionally, the RAPD-PCR analysis revealed a high level of genetic heterogeneity among *P. bovis,* with 18 distinct genotypes distributed randomly across the different farms. Furthermore, *Prototheca* spp. were found to be resistant to most of the tested antimicrobials, except for aminoglycosides (gentamycin, netilmicin, kanamycin), suggesting their potential use as a therapeutic agent for bovine protothecosis. Finally, varying degrees of biofilm production were observed, with most isolates classified as weak producers, followed by moderate producers. Notably, all isolates from the protothecal mastitis outbreak exhibited moderate biofilm production, supporting the notion that biofilm involvement is a key factor in the pathogenicity and infection process of *Prototheca*. Further studies are required to assess the accuracy of aminoglycosides as a therapy against bovine protothecal mastitis and to determine the correlation among biofilm production, *Prototheca* pathogenicity, and antimicrobial resistance.

## Figures and Tables

**Table 1:** Biofilm formation capacity in field isolates and reference strains.

Species	Isolate Type	Biofilm Production	Number of Isolates (%)	OD 490 Range *
*P. bovis*	Field (n = 65)	Moderate	10 (15.4%)	0.176–0.293
		Weak	53 (81.5%)	0.070–0.149
		Non-producer	2 (3.1%)	<0.078
	Reference **	Weak	1 (100%)	0.096
*P. ciferrii*	Field (n = 1)	Weak	1 (100%)	0.081
	Reference ***	Non-producer	1 (100%)	0.075

* OD 490: Optical density at 490 nm after crystal violet staining. The OD 490 range for the negative control was 0.069 to 0.078. ** Reference strain *P. bovis* SAG 2021^T^. *** Reference strain *P. ciferrii* SAG 2063^T^.

## Data Availability

The raw data supporting the conclusions of this article will be made available by the authors on request.

## Abbreviations

The following abbreviations are used in this manuscript:

DNA	Deoxyribonucleic acid
DNAr	Ribosomal DNA
BLASTn	Basic Local Alignment Nucleotide Search tool
CFU	Colony Forming Units
*cytb*	Cytochrome b gene
PCR	Polymerase Chain Reaction
RAPD-PCR	Random Amplified Polymorphic DNA–Polymerase Chain Reaction
SAG	Culture Collection of Algae
MY	Lincomycin
CIP	Ciprofloxacin
ENR	Enrofloxacin
AML	Amoxicillin
P	Penicillin
AMP	Ampicillin
SXT	Sulfamethoxazole/Trimethoprim
CT	Colistin Sulfate
NET	Netilmicin
K	Kanamycin
CN	Gentamicin
ML	Maximum Likelihood Method for phylogenetic inference
BY	Bayesian Inference for phylogenetic inference
UPGMA	Unweighted Pair Group Method with Arithmetic Mean
SDA	Sabouraud Dextrose Agar
SDB	Sabouraud Dextrose Broth
PIM	*Prototheca* Isolation Medium
NMC	National Mastitis Council
CLSI	Clinical and Laboratory Standards Institute

## Appendix A

**Table A1:** Information on each isolate: farm of origin, *Prototheca* species, RAPD-PCR genotype, antimicrobial susceptibility (R: resistant; S: susceptible), and biofilm production (W: weak; M: moderate; N: non-producer), mastitis type (SCM: subclinical mastitis; MC: clinical mastitis), and sample type.

Isolate	Species	Farm	MY	CIP	ENR	AML	P	AMP	SXT	CT	NET	K	CN	Biofilm	RAPD-PCR Genotype	Mastitis Type	Sample Type
1 ORO	*P. bovis*	A	R	R	R	R	R	R	R	S	S	S	S	W	14	SCM	Individual milk
2 ORO	*P. bovis*	A	R	R	R	R	R	R	R	S	S	S	S	W	1	SCM	Individual milk
6 ORO	*P. bovis*	A	R	R	R	R	R	R	R	S	S	S	S	W	15	SCM	Individual milk
3 LP	*P. ciferrii*	B	R	R	R	R	R	R	R	S	S	S	S	W		SCM	Individual milk
4 QUI	*P. bovis*	C	R	R	R	R	R	R	R	R	S	S	S	W	2	MC	Individual milk
5 QUI	*P. bovis*	C	R	R	R	R	R	R	R	R	S	S	S	W	1	MC	Individual milk
11 QUI	*P. bovis*	C	R	R	R	R	R	R	R	S	S	S	S	W	2	CM	Individual milk
12 QUI	*P. bovis*	C	R	R	R	R	R	R	R	R	S	S	S	W	2	SCM	Individual milk
13 QUI	*P. bovis*	C	R	R	R	R	R	R	R	S	S	S	S	N	3	CM	Individual milk
14 QUI	*P. bovis*	C	R	R	R	R	R	R	R	S	S	S	S	W	5	CM	Individual milk
16 QUI	*P. bovis*	C	R	R	R	R	R	R	R	R	S	S	S	W	4	CM	Individual milk
7 SG	*P. bovis*	D	R	R	R	R	R	R	R	S	S	S	S	W	3	CM	Individual milk
8 CAF	*P. bovis*	E	R	R	R	R	R	R	R	S	S	S	S	W	2	-	Individual milk
9 CAF	*P. bovis*	E	R	R	R	R	R	R	R	S	S	S	S	W	1	-	Individual milk
10 COL	*P. bovis*	F	R	R	R	R	R	R	R	S	S	S	S	W	11	CM	Individual milk
15 LL	*P. bovis*	G	R	R	R	R	R	R	R	R	S	S	S	W	9	CM	Individual milk
6 AN	*P. bovis*	H	R	R	R	R	R	R	R	S	S	S	S	W	3	-	Bulk tank milk
7 AN	*P. bovis*	H	R	R	R	R	R	R	R	S	S	S	S	W	3	-	Bulk tank milk
8AN	*P. bovis*	H	R	R	R	R	R	R	R	S	S	S	S	W	1	-	Bulk tank milk
17 AN	*P. bovis*	H	R	R	R	R	R	R	R	S	S	S	S	W	7	SCM	Individual milk
18 AN	*P. bovis*	H	R	R	R	R	R	R	R	S	S	S	S	W	7	CM	Individual milk
19 AN	*P. bovis*	H	R	R	R	R	R	R	R	S	S	S	S	W	2	CM	Individual milk
20 AN	*P. bovis*	H	R	R	R	R	R	R	R	S	S	S	S	W	8	SCM	Individual milk
21 AN	*P. bovis*	H	R	R	R	R	R	R	R	S	S	S	S	W	2	CM	Individual milk
22 AN	*P. bovis*	H	R	R	R	R	R	R	R	S	S	S	S	W	5	CM	Individual milk
23 AN	*P. bovis*	H	R	R	R	R	R	R	R	S	S	S	S	W	3	CM	Individual milk
1LV	*P. bovis*	I	R	R	R	R	R	R	R	S	S	S	S	W	1	-	Bulk tank milk
2LV	*P. bovis*	I	R	R	R	R	R	R	R	S	S	S	S	W	3	-	Bulk tank milk
3LV	*P. bovis*	I	R	R	R	R	R	R	R	S	S	S	S	W	2	-	Bulk tank milk
4 SS	*P. bovis*	J	R	R	R	R	R	R	R	S	S	S	S	W	2	-	Bulk tank milk
5 SS	*P. bovis*	J	R	R	R	R	R	R	R	S	S	S	S	N	6	-	Bulk tank milk
9 SS	*P. bovis*	J	R	R	R	R	R	R	R	S	S	S	S	W	1	-	Bulk tank milk
10 SS	*P. bovis*	J	R	R	R	R	R	R	R	S	S	S	S	W	2	-	Bulk tank milk
11 SS	*P. bovis*	J	R	R	R	R	R	R	R	S	S	S	S	W	5	-	Bulk tank milk
12 PA	*P. bovis*	K	R	R	R	R	R	R	R	R	S	S	S	W	1	-	Bulk tank milk
13 PA	*P. bovis*	K	R	R	R	R	R	R	R	R	S	S	S	W	1	-	Bulk tank milk
14	*P. bovis*	L	R	R	R	R	R	R	R	S	S	S	S	W	5	-	Bulk tank milk
15 CM	*P. bovis*	M	R	R	R	R	R	R	R	S	S	S	S	W	2	-	Bulk tank milk
16 R	*P. bovis*	N	R	R	R	R	R	R	R	R	S	S	S	W	1	-	Bulk tank milk
P4	*P. bovis*	O	R	R	R	R	R	R	R	S	S	S	S	M	3	CM	Individual milk
P12	*P. bovis*	O	R	R	R	R	R	R	R	S	S	S	S	W	1	CM	Individual milk
P15	*P. bovis*	O	R	R	R	R	R	R	R	S	S	S	S	W	3	CM	Individual milk
P18	*P. bovis*	O	R	R	R	R	R	R	R	S	S	S	S	W	1	CM	Individual milk
P19	*P. bovis*	O	R	R	R	R	R	R	R	S	S	S	S	W	2	CM	Individual milk
P21	*P. bovis*	O	R	R	R	R	R	R	R	S	S	S	S	W	1	CM	Individual milk
P22	*P. bovis*	O	R	R	R	R	R	R	R	S	S	S	S	M	2	CM	Individual milk
P23	*P. bovis*	O	R	R	R	R	R	R	R	S	S	S	S	W	1	CM	Individual milk
P25	*P. bovis*	O	R	R	R	R	R	R	R	S	S	S	S	W	15	CM	Individual milk
P26	*P. bovis*	O	R	R	R	R	R	R	R	S	S	S	S	M	2	CM	Individual milk
P30	*P. bovis*	O	R	R	R	R	R	R	R	S	S	S	S	W	1	CM	Individual milk
P31	*P. bovis*	O	R	R	R	R	R	R	R	S	S	S	S	M	13	CM	Individual milk
P33	*P. bovis*	O	R	R	R	R	R	R	R	S	S	S	S	W	1	CM	Individual milk
P35	*P. bovis*	O	R	R	R	R	R	R	R	S	S	S	S	W	2	CM	Individual milk
P38	*P. bovis*	O	R	R	R	R	R	R	R	S	S	S	S	M	16	CM	Individual milk
P39	*P. bovis*	O	R	R	R	R	R	R	R	S	S	S	S	W	2	CM	Individual milk
P40	*P. bovis*	O	R	R	R	R	R	R	R	S	S	S	S	W	1	CM	Individual milk
P42	*P. bovis*	O	R	R	R	R	R	R	R	S	S	S	S	M	2	CM	Individual milk
P43	*P. bovis*	O	R	R	R	R	R	R	R	S	S	S	S	W	1	CM	Individual milk
P45	*P. bovis*	O	R	R	R	R	R	R	R	S	S	S	S	W	2	CM	Individual milk
P47	*P. bovis*	O	R	R	R	R	R	R	R	S	S	S	S	W	10	CM	Individual milk
P49	*P. bovis*	O	R	R	R	R	R	R	R	S	S	S	S	M	18	CM	Individual milk
P53	*P. bovis*	O	R	R	R	R	R	R	R	S	S	S	S	W	17	CM	Individual milk
P54	*P. bovis*	O	R	R	R	R	R	R	R	S	S	S	S	W	16	CM	Individual milk
P55	*P. bovis*	O	R	R	R	R	R	R	R	S	S	S	S	M	13	CM	Individual milk
P58	*P. bovis*	O	R	R	R	R	R	R	R	S	S	S	S	M	12	CM	Individual milk
P59	*P. bovis*	O	R	R	R	R	R	R	R	S	S	S	S	M	13	CM	Individual milk

**Table A2:** GenBank accession numbers of *cytb* partial gene sequences from representative *Prototheca* isolates obtained in the present study. For those sequences with 100% identity among them, only one representative sequence was submitted.

Accession Number	Isolated	Distance Matrix 100%
PV768537	1LV	1ORO; 2LV; 3LV; 4SS; 5SS; 6AN; 6ORO; 7AN; 8AN; 8CAF; 9CAF; 9SS; 10COL; 10SS; 11QUI; 11SS; 13PA; 14BTM; 15CM; 15LL; 16R; 18AN; 20AN; 23AN; P4; P18; P19; P21; P22; P23; P25; P26; P30; P31; P35; P38; P39; P40; P42; P43; P45; P47; P49; P53; P54; P55; P58; P59; 7SG; 12PA; 13QUI; 17AN; 19AN; 22AN
PV768539	5QUI	4QUI; 12QUI; 14QUI; 16ORO
PV768538	P33	
PV768540	21AN	2ORO
PV768541	P12	
PV768542	P15	
PV768543	3LP	
